# Managing Choledocholithiasis in Rare Congenital Hepatobiliary Anomalies: A Report of Three Cases

**DOI:** 10.3390/jcm15197419

**Published:** 2026-09-24

**Authors:** Andreja Ocepek, Luka Strniša, Sabina Vadnjal Đonlagić, Darinka Purg, Sara Nikolić

**Affiliations:** 1 Department of Gastroenterology, University Division of Internal Medicine, University Medical Centre Maribor, Ljubljanska 5, 2000 Maribor, Slovenia; 2 Medical Faculty, University of Maribor, Taborska 8, 2000 Maribor, Slovenia; 3 Clinical Department of Gastroenterology, Division of Internal Medicine, University Medical Centre Ljubljana, Japljeva 2, 1000 Ljubljana, Slovenia; 4 Department of Radiology, University Medical Centre Maribor, Ljubljanska 5, 2000 Maribor, Slovenia

**Keywords:** congenital hepatobiliary anomaly, situs inversus totalis, hepatic lobe anomaly, endoscopic retrograde cholangiopancreatography, case report

## Abstract

**Background**: Endoscopic retrograde cholangiopancreatography is an established therapeutic modality for pancreaticobiliary disease. Congenital anatomical anomalies, including situs inversus totalis and rare hepatobiliary malformations, substantially increase the technical complexity of endoscopic retrograde cholangiopancreatography and are seldom encountered in clinical practice. Published experience is limited predominantly to individual case reports and smaller case series. **Methods**: We present a report of three patients who underwent endoscopic retrograde cholangiopancreatography: two with situs inversus totalis and choledocholithiasis complicated by acute cholangitis, and one with a rare congenital anomaly consisting of a completely separated right hepatic lobe combined with choledocholithiasis. **Results**: Successful endoscopic retrograde cholangiopancreatography was achieved in all three patients. In both situs inversus totalis cases, biliary cannulation and stone clearance were accomplished using modified endoscopic techniques, including endoscope rotation and positional adjustments, without procedural complications. In the patient with the separated hepatic lobe, endoscopic retrograde cholangiopancreatography allowed common bile duct stone clearance; however, an attempted mechanical lithotripsy of a gallbladder calculus resulted in basket entrapment, necessitating surgical treatment. **Conclusions**: Endoscopic retrograde cholangiopancreatography is feasible and effective in patients with congenital hepatobiliary anomalies, but demands meticulous pre-procedural planning, thorough anatomical understanding, and technical versatility. A cautious step-by-step approach and readiness to adapt technique are essential to optimise outcomes and minimise risk.

## 1. Introduction

Congenital anomalies of the liver and biliary tract encompass a broad spectrum of developmental abnormalities, including morphological and positional variants, vascular anomalies, bile duct malformations, and hereditary conditions [1,2]. While many of these anomalies are identified at birth or in early childhood, positional and morphological hepatic variants are often discovered incidentally on imaging in adulthood [1]. Knowledge of such variants is of critical importance when planning surgical or endoscopic interventions.

Situs inversus totalis (SIT) is a rare congenital condition characterised by complete mirror-image transposition of all thoracic and abdominal viscera along the left–right axis. Its prevalence in the general population is estimated at 1 in 5000 to 1 in 25,000 live births [3,4]. The condition follows an autosomal recessive inheritance pattern and has been linked to mutations in genes regulating left–right axis patterning during embryogenesis, particularly genes associated with primary ciliary dyskinesia [4]. Isolated SIT is typically asymptomatic, and affected individuals lead normal lives. However, the reversed anatomy poses significant challenges in emergency and elective medical interventions [4].

Endoscopic retrograde cholangiopancreatography (ERCP) is one of the most technically demanding procedures in gastroenterology. In patients with normal anatomy, it is routinely performed with the patient in the left lateral decubitus or left prone position, an approach that is aligned with standard organ orientation. In patients with SIT, this positioning may be suboptimal or counterproductive, as the reversed visceral anatomy fundamentally alters the endoscopic view, the orientation of the major papilla, and the direction of biliary cannulation. The American Society for Gastrointestinal Endoscopy (ASGE) classifies ERCP in surgically altered anatomy as a Level IV (most complex) procedure; by analogy, ERCP in naturally altered anatomy, such as SIT, represents an equivalent degree of difficulty [4]. Published experience with ERCP in SIT consists almost exclusively of case reports and small case series, making it one of the least standardised areas of therapeutic endoscopy.

Beyond SIT, rare congenital morphological anomalies of the liver, including ectopic or accessory hepatic lobes, hepatic lobe agenesis, and complete lobar separation, may profoundly alter the anatomy of the biliary tree and further complicate endoscopic or surgical access. Such anomalies are exceptionally rare, and their management during ERCP has not been systematically described in the literature.

This case series presents three patients with congenital hepatobiliary anomalies who underwent ERCP: two patients with SIT and acute cholangitis, and one patient with a unique congenital anomaly consisting of complete separation of the right hepatic lobe combined with choledocholithiasis. The clinical features, technical challenges, procedural adaptations, and outcomes are described in detail.

## 2. Materials and Methods

This is a report of three cases from two tertiary referral centres in Slovenia (University Medical Centre Maribor and Ljubljana). All three patients presented with biliary indications for ERCP and were found to have congenital hepatobiliary anomalies. Clinical, laboratory, radiological, and procedural data were retrieved from electronic medical records. All procedures were performed by experienced endoscopists (each with more than 1000 independent ERCP procedures). Standard Olympus side-viewing duodenoscopes were used in all cases. Reporting follows the CARE (Case Report). 

In all patients, pre-procedural cross-sectional imaging (computed tomography [CT] and/or magnetic resonance cholangiopancreatography [MRCP]) was performed to delineate the relevant anatomy before ERCP. Procedural details, including patient and endoscopist positioning, scope manipulation technique, cannulation method, therapeutic interventions, and outcomes, are reported for each case. Acute cholangitis was graded according to the Tokyo Guidelines 2018 severity criteria [5]. Procedural complications were defined and classified according to the European Society for Gastrointestinal Endoscopy (ESGE) 2020 criteria [6].

## 3. Results

### 3.1. Clinical Case 1

A 77-year-old woman with known dextrocardia, thoracic aortic aneurysm, ischaemic cardiomyopathy, chronic atrial fibrillation, and chronic kidney disease was admitted with acute cholangitis. Her symptoms included intense epigastric pain, vomiting, and jaundice. Laboratory investigations demonstrated direct hyperbilirubinaemia, cholestatic enzyme elevation, leukocytosis, and elevated C-reactive protein (CRP) and procalcitonin (PCT) levels. Abdominal ultrasound, CT, and MRCP confirmed cholelithiasis and a 7 mm calculus in the distal common bile duct (CBD), together with SIT (Figure 1a). Acute cholangitis grade I (mild) according to Tokyo Guidelines 2018 was diagnosed. Empirical antibiotic therapy with cefuroxime and metronidazole was initiated, and anticoagulant therapy was temporarily discontinued before ERCP. Following a review of available literature on ERCP in SIT, the procedure was performed five days after admission with the patient in the standard left lateral decubitus position, with the endoscopist standing on the right side of the examination table. An initial diagnostic upper gastrointestinal endoscopy was performed to familiarise the operator with the reversed anatomy before introducing the duodenoscope. The duodenoscope was advanced using the technique of two sequential left-turn rotations during insertion. After careful withdrawal and detailed examination of the duodenum, the major papilla was visualised in its expected location, at 1–3 o’clock. Initial cannulation with a standard sphincterotome failed; CBD cannulation was subsequently achieved using a standard cannula with a guidewire. Correct biliary placement was confirmed by fluoroscopy and bile aspiration (Figure 1b). Contrast cholangiography demonstrated the CBD calculus. Endoscopic sphincterotomy (EST) was performed, and the stone was removed successfully with an extraction balloon (Figure 1c,d). No immediate procedural complications occurred, four days following ERCP bilirubin levels normalized. The patient recovered uneventfully; inflammation resolved with antibiotic therapy, and multidisciplinary consultations for elective cholecystectomy were initiated. At discharge, six days after ERCP, the patient reported feeling well and expressed relief that the anatomical variant had not prevented a minimally invasive treatment option.

### 3.2. Clinical Case 2

A 93-year-old man with a background of type 2 diabetes mellitus, arterial hypertension, chronic kidney disease, bilateral hip prostheses, and known gallstones was admitted with acute cholangitis. Laboratory findings showed direct hyperbilirubinaemia, cholestatic enzyme elevation, CRP 271 mg/L, leukocytosis, and elevated blood urea nitrogen. CT demonstrated small gallbladder stones and a probable stone in the distal CBD (Figure 2a); the CBD measured 12 mm in diameter with wall thickening, consistent with acute cholangitis grade II (moderate) according to Tokyo Guidelines 2018. Known SIT was confirmed. Given the severity of cholangitis and the patient’s age, day after admission urgent ERCP was performed. The procedure was carried out with the patient in the standard left lateral decubitus position, with the endoscopist standing on the right side of the examination table. A 180-degree counterclockwise rotation of the duodenoscope in the stomach was used to navigate past the pylorus into the duodenum. Once in the second part of the duodenum, the scope was shortened in the opposite direction to the standard manoeuvre, enabling front-face visualisation of the major papilla. A standard sphincterotome and guidewire were used for CBD cannulation, confirmed by fluoroscopy (Figure 2b). Endoscopic sphincterotomy was performed, followed by extraction of multiple small microliths using a balloon catheter. Due to the patient’s advanced age and the complexity of ductal clearance, a biodegradable biliary stent (ARCHIMEDES^TM^ 10 Fr, 60 mm, Q3 Medical Germany GmbH, Winsen, Germany) was placed to ensure ongoing biliary drainage (Figure 2c,d) and to preclude follow-up endoscopy for stent removal. The procedure was completed without complications. Antibiotic therapy and supportive measures were continued. Three days after ERCP bilirubin levels normalized and CRP showed a marked decline. Given the patient’s advanced age and overall condition, surgical intervention was not planned. At 93 years of age, the patient was relieved to avoid surgery, and reported feeling well with a restored appetite before being discharged.

### 3.3. Clinical Case 3

A 59-year-old woman with a history of longstanding biliary colic during past six years was referred for elective ERCP for choledocholithiasis. She had no history of prior ERCP. Contrast-enhanced CT of the abdomen described a rare congenital hepatic anomaly: a completely separated right hepatic lobe in its normal anatomical position, and a left hepatic lobe located caudal to the duodenum, connected to the right lobe by a dilated bile duct (Figure 3a,b). Choledocholithiasis (an 18 mm CBD calculus) and cholecystolithiasis were identified. Three months prior elective cholecystectomy had been abandoned due to the altered anatomy. 

ERCP was performed in the standard left lateral decubitus position. Owing to the aberrant biliary anatomy, CBD cannulation was initially challenging but was ultimately achieved with a sphincterotome and guidewire manipulation. Fluoroscopic contrast cholangiography demonstrated a markedly dilated CBD with an 18 mm calculus and complex ductal anatomy: the CBD initially coursed towards the left hepatic lobe, with a wide communicating branch to the right lobe visible (Figure 4a). Endoscopic sphincterotomy, balloon dilation of the papilla, and mechanical lithotripsy were performed with successful fragmentation and extraction of the CBD calculus (Figure 4b). 

During re-engagement with the wire following stone clearance, the guidewire inadvertently passed through the cystic duct into the gallbladder and was confirmed fluoroscopically. Basket lithotripsy of the gallbladder calculus was attempted, but resulted in basket fracture and entrapment within the gallbladder (Figure 5a,b), constituting a severe procedural complication according to ESGE 2020 criteria and necessitating surgical treatment [6]. 

Rather than withdrawing the guidewire and concluding the procedure after CBD stone extraction, which was the primary therapeutic objective, a decision was made intraoperatively to attempt transpapillary basket lithotripsy of the gallbladder calculus. In retrospect, this represented a deviation from standard management. The correct approach at this time point would have been to withdraw the guidewire, end the procedure with CBD clearance as the sole endpoint, and refer the patient for elective multidisciplinary reassessment, involving gastroenterology, hepatobiliary surgery, and radiology, to plan definitive gallbladder management in the context of her rare anatomical variant. The contextual factors that influenced the decision, the patient’s known cholecystolithiasis, the prior deferred cholecystectomy, and the inadvertent availability of cystic duct access, are acknowledged, but do not constitute adequate argumentation for proceeding with a non-standard intervention. The patient was transferred to the operating room and open cholecystectomy with removal of the foreign body was performed. The postoperative course was uneventful. Despite the procedural complication, the patient was gratified that treatment was ultimately successful and that she was able to undergo cholecystectomy, which she had previously been denied due to the anatomical challenges posed by her condition.

Summary of the three cases is presented in Table A1 (Appendix A).

## 4. Discussion

This case series illustrates the challenges and potential pitfalls of ERCP in patients with rare congenital hepatobiliary anomalies, focusing primarily on SIT and a unique congenital hepatic malformation. Across our three cases, ERCP was technically feasible and therapeutically successful in achieving its primary objectives; however, the procedures required substantial modifications in technique, pre-procedural planning, and intra-procedural decision-making.

### 4.1. ERCP In Situs Inversus Totalis: Technical Considerations

SIT fundamentally alters the endoscopic landscape during ERCP. The reversal of visceral anatomy affects the orientation of the duodenum, the position and angulation of the major papilla, and the direction of the biliary orifice. In normal anatomy, the biliary orifice is oriented at approximately the 10–11 o’clock position; in SIT, this is inverted to approximately the 1–3 o’clock position, requiring the cannulation direction to be performed in mirror image relative to standard technique [7,8]. Furthermore, scope shortening in the duodenum may be technically more demanding when the endoscope has been rotated through 180 degrees, as this creates loop instability and reduces control of the scope tip [4,7]. The published literature on ERCP in SIT consists almost entirely of case reports and one multicentre retrospective case series [7]. In the largest study to date, a multicenter Chinese series of 14 SIT patients who underwent ERCP, successful selective biliary cannulation was achieved in 85.7% of cases (12/14), with difficult cannulation encountered in 71.4% [7]. Procedural complications occurred in 21.4% of patients (bleeding, pneumonia, and acute myocardial infarction in one patient each), with a notably higher complication rate in patients maintained in the prone position compared to those in modified positions (33.3% versus 18.2%) [7]. These findings highlight that even in experienced hands, ERCP in SIT carries a substantially higher rate of technical difficulty and adverse events than in patients with normal anatomy. No universally accepted standard for patient or endoscopist positioning during ERCP in SIT has been established [9,10]. The most commonly described approaches in the literature include:

- The prone position with the endoscopist on the patient’s right side, using a 180-degree clockwise rotation of the endoscope in the stomach [10,11,12,13];
- The “mirror image” technique, where the patient is placed in the right lateral decubitus position and all endoscopic manoeuvres are performed inversely [4,11];
- The supine position with the endoscopist on the left side, which mimics the organ orientation of a normal patient in the prone position [7,8,14];
- A 360-degree rotation technique involving a sequential 180-degree rotation in the stomach followed by a further 180-degree rotation in the duodenum [4].

Rotatable sphincterotomes have been reported to facilitate cannulation in several cases by allowing independent angulation of the cutting wire relative to the biliary orifice [12,13,15]. Additionally, adjustment of the fluoroscopic monitor position to the patient’s foot side has been described as a means of preserving standard operator posture without necessitating patient repositioning [15].

In all three cases, we opted for the standard left lateral decubitus position. ERCP in SIT patients preceded by a diagnostic upper gastrointestinal endoscopy to allow the operator to familiarise herself with the reversed anatomy before proceeding with the therapeutic procedure. This approach, which has been reported by others, offers the advantage of an unaltered endoscopic room setup and allows the endoscopist to apply their standard handling habits while mentally adjusting to the inverted view [8,16]. In Case 1, when initial cannulation with a standard sphincterotome failed, a switch to a standard cannula with a guidewire, a recognised rescue technique, enabled successful deep biliary access [7]. In Cases 1 and 2, a 180-degree counterclockwise rotation in the stomach combined with scope shortening in the opposite direction was employed, consistent with approaches described by Fiocca et al. and others [10,11,12,17]. The use of a biodegradable biliary stent, although not recommended standard of care, at the end of the procedure in case 2 (a 93-year-old patient) was a pragmatic decision to ensure sustained biliary drainage given his age and comorbidities, while avoiding the need for a further elective stent retrieval procedure [18,19]. The key lesson from both SIT cases aligns with the consensus emerging from the literature: there is no single optimal technique, and the endoscopist’s familiarity with the chosen approach is of greater importance than the specific technique selected [9,10,11]. Pre-procedural review of imaging to understand the specific anatomy, a willingness to adapt technique intra-procedurally, and the availability of adjunctive tools such as rotatable sphincterotomes and dual-guidewire techniques are essential prerequisites for success [4,14].

### 4.2. Complications of ERCP in SIT

Complications of ERCP in SIT appear to be infrequent in the published literature, though the small sample sizes preclude definitive conclusions. In the multicentre series by Ding et al., the overall adverse event rate was 21.4%, with no post-procedural pancreatitis or deaths [7]. One case of post-sphincterotomy bleeding following transpancreatic precut was managed conservatively. A case of bleeding from portal biliopathy during stone removal in a SIT patient was treated with self-expandable metal stent placement and balloon compression [7]. It is also noteworthy that the direction of sphincterotomy in SIT, from the 1–3 o’clock rather than the 10–12 o’clock position, may alter the risk profile of post-sphincterotomy bleeding, given that the vascular distribution around the papilla may also be inverted [7]. Neither of our two SIT cases experienced procedural complications, consistent with the general feasibility and safety of ERCP in SIT when performed by experienced operators [8,14].

### 4.3. ERCP in Congenital Hepatic Lobe Anomalies

Case 3 represents a qualitatively different clinical challenge. Rare congenital morphological anomalies of the liver, including ectopic hepatic lobes, accessory lobes, hepatic lobe hypoplasia or aplasia, and, as in this case, complete anatomical separation of the right and left hepatic lobes, can fundamentally alter the architecture of the biliary tree and the course of the CBD. In our patient, the left hepatic lobe was displaced caudally beneath the duodenum, connected to the anatomically normal right lobe by a dilated communicating bile duct. This configuration created a complex, angulated biliary anatomy that challenged both cannulation and therapeutic manoeuvres. The closest analogies in the literature are cases of ectopic or accessory hepatic lobes and Riedel’s lobe variants, which are themselves rare findings [20,21,22]. Choledocholithiasis in the context of congenital hepatic anomalies has been reported in association with choledochal cysts and Caroli’s disease, but not in the specific configuration seen here [18].

The decision to attempt transpapillary basket lithotripsy of the gallbladder calculus in Case 3, following inadvertent cystic duct access during CBD stone clearance, requires careful contextualisation. Transpapillary endoscopic access to the gallbladder, including transpapillary drainage and stone extraction, is an established although technically demanding procedure, reported with technical success rates of 70–90% in selected series [23,24]. Its use is recognised in patients with acute cholecystitis unsuitable for surgery, and its feasibility for stone extraction in patients with concomitant choledocholithiasis and cholecystolithiasis who decline or are unfit for cholecystectomy has been described [25,26]. In our patient, several factors converged to guide the endoscopist to attempt gallbladder stone clearance at the time of CBD clearance: (1) the gallbladder calculus was a clinically relevant finding with a high likelihood of future complications, since untreated cholecystolithiasis produces symptomatic events or biliary complications in up to 20% of patients over 10 years [27]; (2) the altered anatomy had precluded cholecystectomy before, making future surgical access uncertain; (3) transpapillary cystic duct access had been inadvertently established and was patent; and (4) the gallbladder calculus was potentially amenable to basket fragmentation and clearance given the dilated cystic duct. The above factors reflect the intraoperative rationale as perceived at the time; however, in retrospect they are insufficient to justify proceeding with a non-standard intervention. As detailed in the case report, the appropriate management at this point would have been to terminate the procedure after CBD clearance and refer the patient for elective multidisciplinary planning. The attempt is therefore retrospectively assessed as a deviation from standard practice, described here as an instructive example of the risks of extending unplanned manoeuvres beyond the defined procedural endpoint. 

Procedural decision-making at the time was also supported by dedicated surgical backup planned prior to ERCP. The attempt was associated with significant inherent risk. The use of a standard extraction basket within the gallbladder carries substantially higher risk of entrapment than use within the CBD. Basket entrapment in the CBD during standard mechanical lithotripsy has an incidence of approximately 0.2–0.5% [28]. When a basket is deployed outside its validated anatomical target, as in an acutely angulated cystic duct, the gallbladder fundus, or the pancreatic duct, the risk of entrapment rises considerably, because the basket cannot be opened or closed in a controlled plane and the wire geometry is unfavourable for stone fragmentation or safe basket release [28,29]. No major endoscopy society guideline specifically endorses transpapillary mechanical basket lithotripsy within the gallbladder as a standard technique. The established transpapillary gallbladder approach involves guidewire access, cystic duct dilation, and stent or drainage catheter placement, not basket lithotripsy in the gallbladder cavity itself [23,30]. When intraductal lithotripsy of gallbladder or cystic duct stones is undertaken endoscopically, the preferred approach, where expertise is available, is cholangioscopy-directed electrohydraulic or laser lithotripsy, which allows direct visualisation of the stone and avoids blind basket deployment [31,32]. In retrospect, once inadvertent gallbladder access was confirmed fluoroscopically and the decision to attempt stone clearance was taken, the safer intra-procedural approach would have been basket-free extraction (balloon catheter dislodgement of the stone into the CBD followed by balloon clearance) or placement of a transpapillary gallbladder stent as a bridge to a subsequent session using cholangioscopy-assisted lithotripsy, rather than direct basket deployment into the gallbladder cavity [32,33,34]. This case therefore illustrates not only the risk of extending unplanned manoeuvres in aberrant anatomy, but specifically the importance of technique selection when gallbladder access is established. ESGE guidelines advise a cautious, stepwise approach in anatomically complex cases, and recommend that additional therapeutic interventions beyond the planned procedure be carefully considered before undertaking them [6,11]. The basket fracture in this case was managed by prompt surgical activation with open cholecystectomy rather than a laparoscopic approach, which was dictated by the abnormal anatomy and the need for secure intraoperative identification of all biliary structures. This reflects the broader principle that in patients with rare congenital hepatobiliary anomalies, surgical planning must be individualised and conventional approaches cannot be assumed to be applicable [35]. Finally, it is important to acknowledge, that even had basket lithotripsy of the gallbladder calculus succeeded, cholecystectomy would still have been recommended to eliminate the risk of future complications (cholecystitis, Mirizzi syndrome, malignancy). Transpapillary gallbladder stone removal should therefore in most cases not be considered a definitive alternative to cholecystectomy, but at most a temporary solution of acute complications in patients in whom surgery is deferred [26,36].

### 4.4. General Principles for ERCP in Congenital Hepatobiliary Anomalies

The technical complexity of ERCP in congenital hepatobiliary anomalies stems from two distinct but related challenges: altered spatial anatomy and altered perceptual-motor mapping. Understanding how to address each systematically is more instructive than a generic checklist of precautions. The first challenge is anatomical prediction: determining, before the procedure begins, the expected orientation of the papilla, the likely cannulation vector, and the course of the biliary tree. In SIT, the fundamental consequence of mirror-image reversal is that the biliary orifice, normally situated at the 10–11 o’clock position, is displaced to approximately the 1–3 o’clock position [4,9]. This inversion is not merely a detail, it determines the direction of the sphincterotome tip during cannulation attempts, the axis of sphincterotomy, and the risk profile of post-sphincterotomy bleeding. Pre-procedural coronal and axial MRCP reconstructions should be used not simply to identify stones, but to mentally simulate the cannulation geometry. The endoscopist should trace the expected CBD axis on imaging, note the angular relationship between the CBD and the duodenal lumen, and identify any additional anatomical challenges such as periampullary diverticula or an ectopic papilla, both of which have been reported in SIT and further compound cannulation difficulty [7,8,14]. Emerging evidence from simulation studies suggests that three-dimensional (3D) visualisation of biliary anatomy may meaningfully improve cannulation performance by allowing the operator to internalise the spatial geometry of the ductal system before approaching the papilla [37,38]. While validated specifically in ERCP training, the principle is directly applicable to pre-procedural planning in anatomically complex cases. Reviewing 3D biliary reconstructions generated from MRCP data allows the endoscopist to mentally rehearse the approach vector, anticipate loop formation during scope insertion, and pre-select the appropriate device. For a separated hepatic lobe variant such as encountered in Case 3, 3D reconstruction is particularly valuable, as conventional axial imaging may not adequately convey the spatial relationship between a caudally displaced lobe and the CBD, nor the angulation of the communicating duct that will determine whether therapeutic instruments can be directed into it. The second challenge is perceptual-motor translation—the cognitive task of converting standard hand movements into their mirror-image equivalents in real time during the procedure. This “mental translation” problem is specific to SIT and has no analogue in standard ERCP or in most other forms of surgically altered anatomy. When the endoscopist rotates the duodenoscope clockwise in a patient with SIT, the scope tip moves in the opposite direction relative to the visual field compared with normal anatomy. Similarly, upward tip deflection may produce a caudal rather than cranial effect on the endoscopic image, depending on how the scope has been rotated during insertion. The most reliable strategy for managing this perceptual inversion is to choose a patient and endoscopist positioning that minimises the degree of cognitive remapping required. The supine position, reported by others, aligns organ orientation in the SIT patient with that of a normal patient in the prone position, thereby allowing the endoscopist to apply standard manoeuvres without mental reversal. However, in the supine position, adequate airway protection is mandatory. When the prone or lateral position is used instead, the operator must consciously invert the directionality of all rotational and tip-deflection movements. Adjusting the fluoroscopic and endoscopic monitors to the patient’s foot side, as described by Zennyoji et al., is a practical modification that allows the operator to maintain standard body posture while viewing an image that corresponds to the reversed anatomy, reducing the cognitive load of simultaneous scope manipulation and visual reorientation [14]. 

For the separated hepatic lobe variant, the relevant decision-making algorithm differs substantially from that applicable to SIT. Here, the primary intraoperative risk is not perceptual inversion but unanticipated biliary architecture. Because the communicating duct between the two hepatic lobes may subtend an acute angle relative to the CBD, instruments advanced beyond the primary cannulation site may enter an unexpected ductal territory. Guidewire passage through the cystic duct into the gallbladder is common during ERCP and usually inconsequential. In some cases the wire repeatedly enters the cystic duct when it is being directed towards the proximal common bile duct. Cholangiography can then be used to image the cystic duct take-off, the angle of approach can be altered by changing the position of the endoscope in the duodenum and by angling the tip of the papillotome, and where an angled-tip guidewire is used, rotating the wire can direct it into the proximal bile duct [39]. The key principle, underscored by the complication in Case 3, is that any passage of a wire or device beyond the primary target must be preceded by fluoroscopic confirmation of position. The decision to extend therapeutic manoeuvres to unplanned and non-standard procedures, such as the gallbladder stone lithotripsy in Case 3, should be treated as a new procedural decision requiring reassessment of the risk–benefit balance in the context of the aberrant anatomy, not as a routine opportunistic step. In practice, this means pausing after achieving the primary therapeutic goal, reviewing the fluoroscopic images, and explicitly deciding whether additional interventions are appropriate given the anatomical context, the devices available and the high degree of possible complications. Taken together, these considerations argue for a structured pre-procedural algorithm specific to congenital hepatobiliary anomalies:

(1) Obtain coronal 3D MRCP reconstruction and use it to simulate cannulation geometry and identify aberrant ductal branching;
(2) Select patient positioning based on which option minimises perceptual-motor remapping for the individual operator;
(3) Define the therapeutic objectives explicitly before beginning, and treat any extension beyond those objectives as a separate decision requiring fluoroscopic re-assessment;
(4) Have rotatable sphincterotome since it is disproportionately useful in this context;
(5) Plan surgical contingency before the procedure, not reactively afterwards.

Formal procedural complexity grading by international endoscopy societies, acknowledging SIT and rare hepatic morphological anomalies as Level IV procedures, would support appropriate referral and resource allocation. As Aujla et al. noted, explicit complexity classification would facilitate referral of these patients to high-volume specialist centres and underpin adequate informed consent [4].

## 5. Conclusions

ERCP is a feasible and effective therapeutic modality in patients with congenital hepatobiliary anomalies, including SIT and rare morphological hepatic malformations. However, these conditions substantially increase technical complexity and procedural risk. Success depends on thorough pre-procedural anatomical assessment, operator experience, flexibility of technique, and disciplined restraint in limiting interventions to those that are clearly planned and anatomically appropriate. The complication encountered in Case 3 serves as a reminder that in aberrant biliary anatomy, the risks of extending therapeutic manoeuvres beyond the primary indication must be carefully weighed. Larger prospective studies and multi-centre registries are needed to establish evidence-based guidance for ERCP in these challenging clinical scenarios.

## Figures and Tables

**Figure 1:**
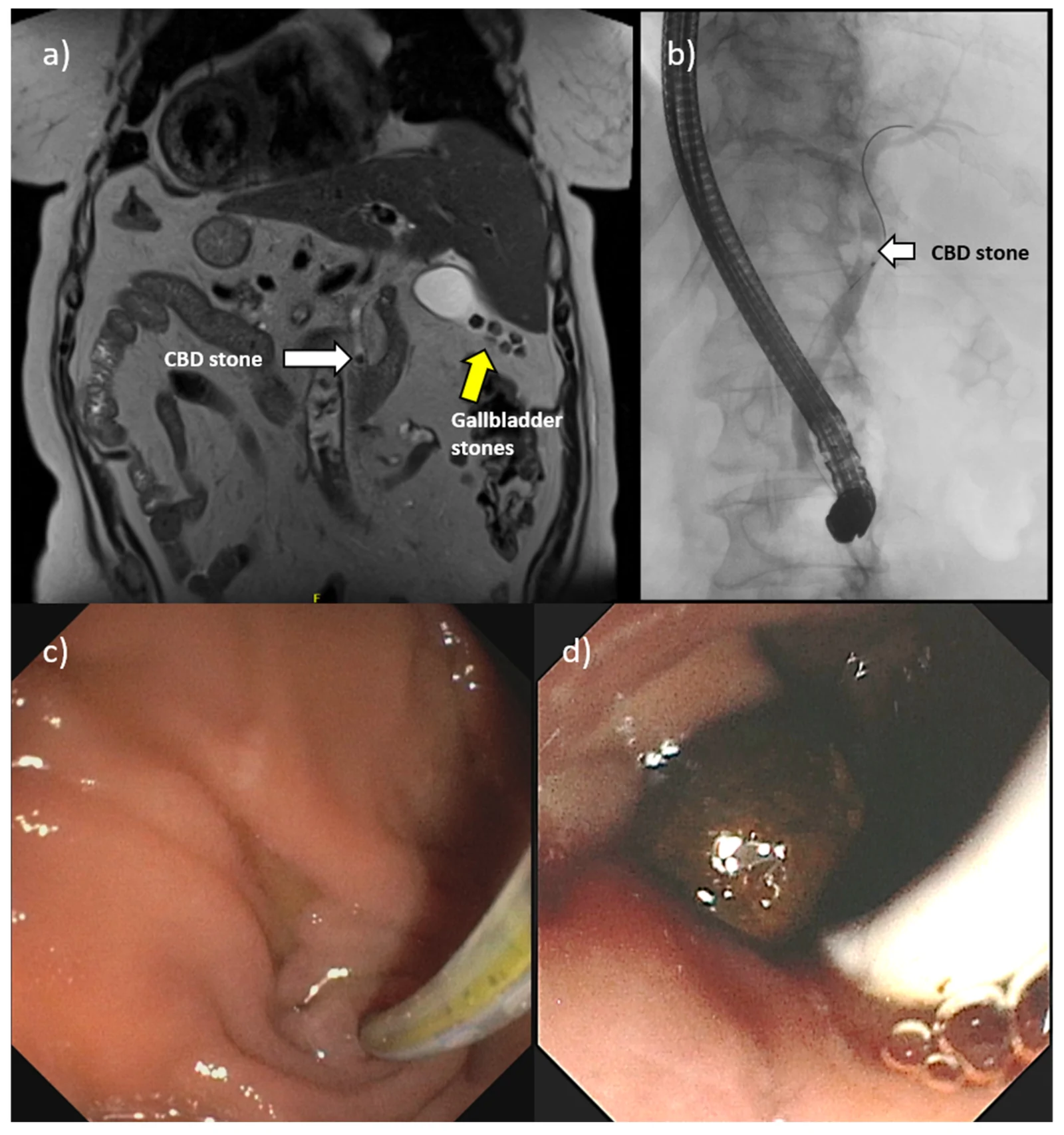
(**a**) Mmagnetic resonance cholangiopancreatography (MRCP) showing situs inversus totalis (SIT), choledocholithiasis (white arrow) and cholecystolithiasis (yellow arrow); (**b**) cholangiography during endoscopic retrograde cholangiopancreatography (ERCP) showing biliary stone inside common bile duct (white arrow); (**c**) endoscopic view of papilla with bile aspiration confirming cannulation of common bile duct (CBD); (**d**) endoscopic view of extracted brown stone.

**Figure 2:**
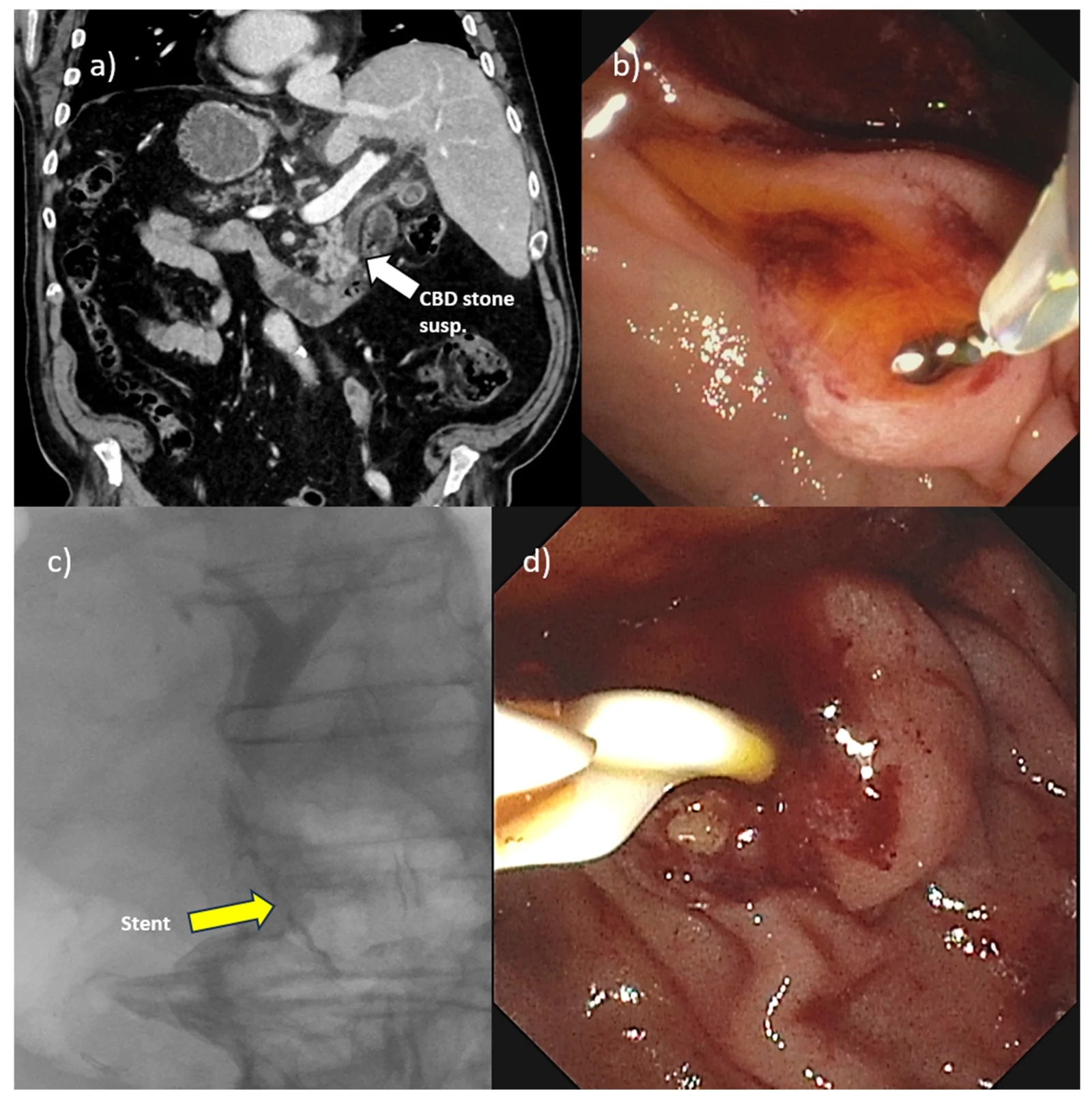
(**a**) Abdominal computed tomography (CT) scan showing situs inversus totalis (SIT) and choledocholithiasis (white arrow); (**b**) endoscopic view of papilla during endoscopic retrograde cholangiopancreatography (ERCP) with guidewire cannulation; (**c**) cholangiography with placed biodegradable biliary stent (yellow arrow); (**d**) endoscopic view of the ARCHIMEDES^TM^ biodegradable biliary stent in situ at the papilla.

**Figure 3:**
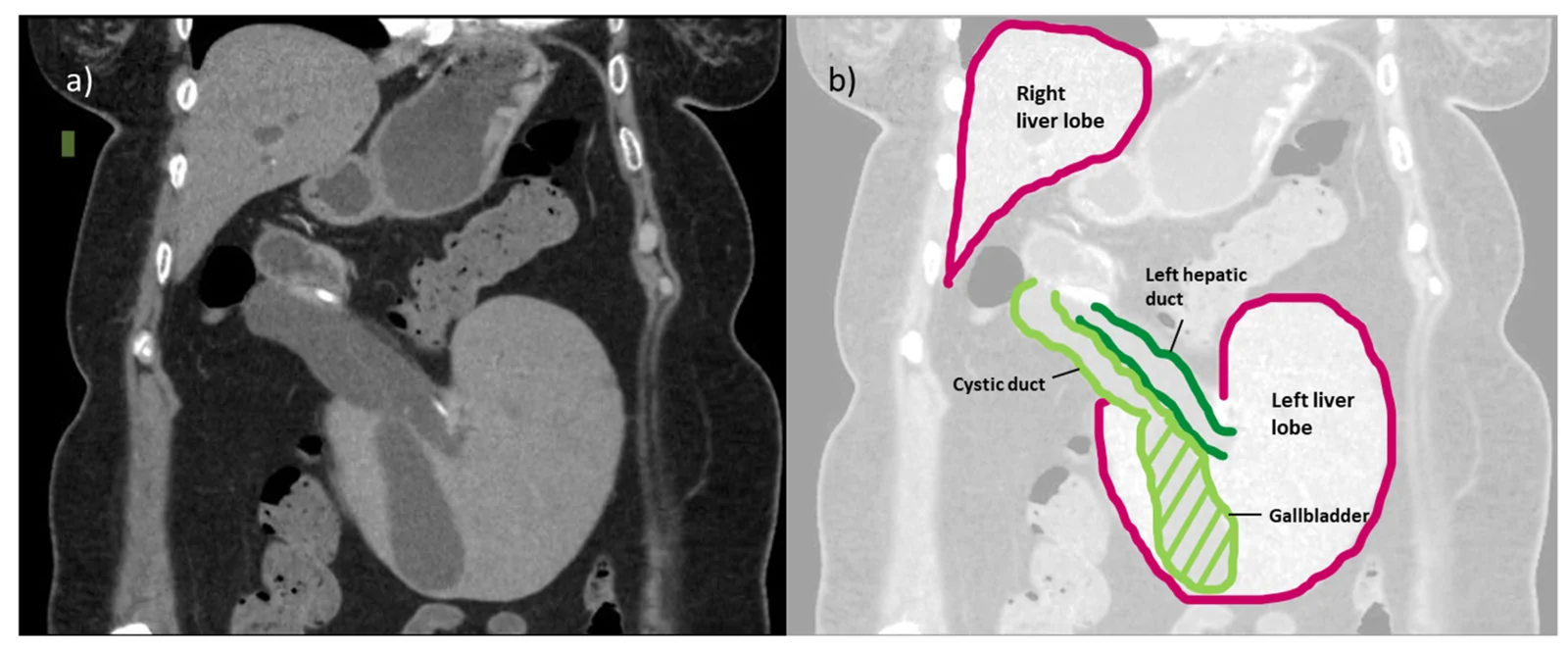
(**a**) Abdominal computed tomography (CT) scan showing hepatobiliary anomaly with completely separated left liver lobe displaced caudally; (**b**) schematic representation of the CT with annotations of anatomical structures: left and right liver lobes (dark red border), gallbladder (light green border with lines), cystic duct (light green border) and left hepatic duct (dark green border).

**Figure 4:**
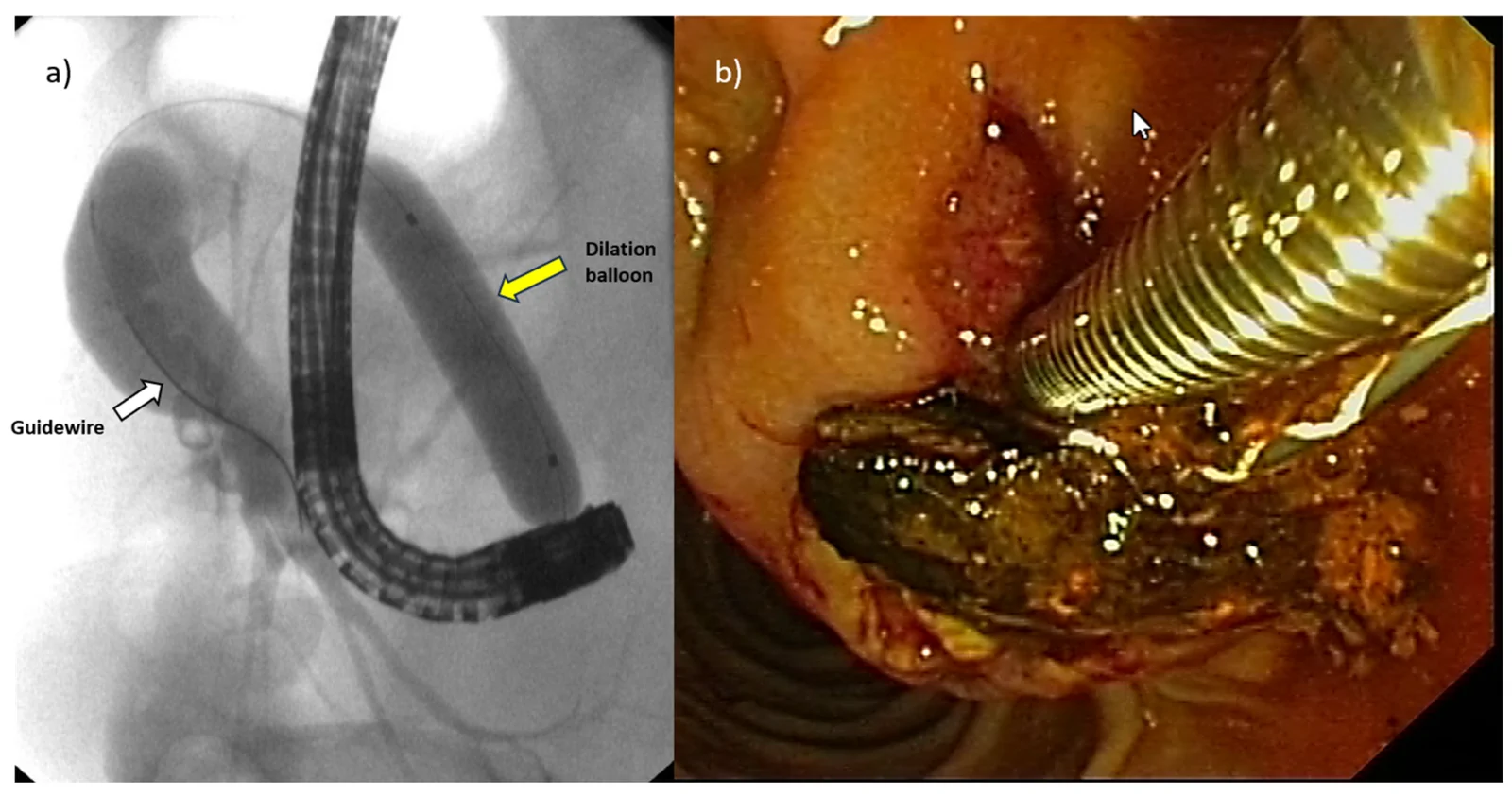
(**a**) Cholangiography during balloon sphincteroplasty (yellow arrow) with the guidewire (white arrow) in the communicating branch of the right hepatic duct; (**b**) endoscopic view of extracted stone fragment, guidewire and lithotriptor in situ.

**Figure 5:**
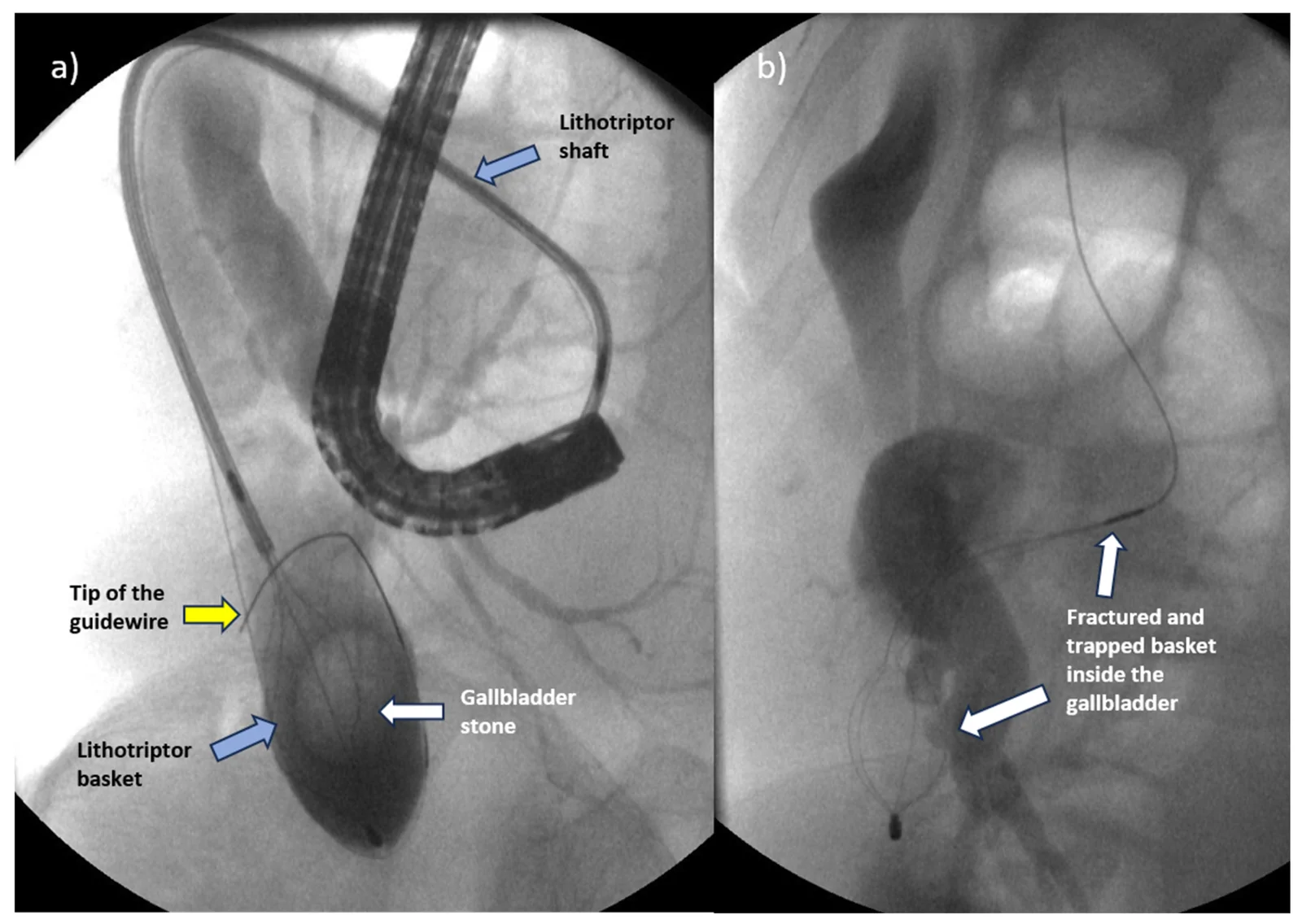
(**a**) Cholangiography showing gallbladder stone (white arrow), tip of the guidewire inside the gallbladder (yellow arrow) and mechanical lithotriptor shaft and basket (blue arrows) in situ; (**b**) cholangiography showing fractured lithotriptor basket entrapped within the gallbladder (white arrows).

## Data Availability

The data presented in this study are available on reasonable request from the corresponding author due to patients’ privacy and ethical reasons.

## Abbreviations

The following abbreviations are used in this manuscript:

SIT	Situs inversus totalis
ERCP	Endoscopic retrograde cholangiopancreatography
ASGE	The American Society for Gastrointestinal Endoscopy
CARE	Case Report
CT	Computed tomography
MRCP	Magnetic resonance cholangiopancreatography
ESGE	European Society for Gastrointestinal Endoscopy
CRP	C-reactive protein
PCT	Procalcitonin
CBD	Common bile duct
EST	Endoscopic sphincterotomy

## Appendix A

**Table A1:** Summary of clinical cases, procedural details, and outcomes.

Category	Variable	Case 1	Case 2	Case 3
Patient and clinical characteristics				
	Age/Gender	77 years/Female	93 years/Male	59 years/Female
	Congenital anomaly	Situs inversus totalis (SIT)	Situs inversus totalis (SIT)	Complete separation of right and left hepatic lobes; right lobe displaced caudal to duodenum
	Primary ERCP indication	Acute cholangitis (CBD stone)	Acute cholangitis (CBD stones)	Choledocholithiasis + cholecystolithiasis
	Cholangitis severity (TG18)	Grade I (mild)	Grade II (moderate)	N/A (elective)
	Relevant comorbidities	Thoracic aortic aneurysm, ischaemic cardiomyopathy, AF, CKD	Type 2 DM, arterial hypertension, CKD, bilateral hip prostheses	None significant; prior defered elective cholecystectomy (due to anatomy)
Pre-procedural workup				
	Imaging modality	Ultrasound, CT, MRCP	Contrast-enhanced CT	Contrast-enhanced CT
	Key anatomical findings	SIT confirmed; 7 mm distal CBD calculus; gallbladder with stones in situ	SIT confirmed; CBD 12 mm with wall thickening; multiple microliths in gallbladder; CBD stone probable	Completely separated hepatic lobes; left lobe caudal to duodenum; 18 mm CBD calculus; gallbladder calculus
ERCP technique				
	Patient position	Left lateral decubitus	Left lateral decubitus	Left lateral decubitus
	Endoscopist position	Right side of the table	Right side of the table	Right side of the table
	Scope manipulation	Two sequential left-turn rotations during insertion; diagnostic upper GI endoscopy performed first to familiarise operator with reversed anatomy	180° counterclockwise rotation in stomach; scope shortened in opposite direction to standard to achieve front-face papilla view	Standard insertion; cannulation challenging due to aberrant ductal anatomy
	Cannulation method	Standard sphincterotome + guidewire; initial failure → rescue with standard cannula + guidewire	Standard sphincterotome + guidewire	Standard sphincterotome + guidewire; initially challenging
	Difficult cannulation	Yes (initial failure with sphincterotome)	No	Yes (aberrant biliary architecture)
	Biliary orifice orientation	Inverted (1–3 o’clock)	Inverted (1–3 o’clock)	Standard orientation (anatomy-specific challenge from ductal course)
Therapeutic interventions				
	Endoscopic sphincterotomy	Yes	Yes	Yes
	Balloon papilla dilation	No	No	Yes
	Stone extraction method	Balloon extraction catheter	Balloon extraction catheter	Mechanical lithotripsy (18 mm stone fragmentation) + balloon extraction
	Stone size/number	7 mm/single	Multiple microliths	18 mm/single (CBD); gallbladder calculus also present
	Complete CBD clearance	Yes	Yes	Yes
	Additional intervention	None	Biodegradable biliary stent placed (to ensure ongoing drainage; avoid repeat ERCP for stent removal)	Attempted transpapillary basket lithotripsy of gallbladder calculus following inadvertent cystic duct access (non-standard; see text)
Outcomes				
	Technical success	Yes	Yes	Partial (CBD cleared; gallbladder intervention complicated)
	Procedural complication	None	None	Basket fracture and entrapment within gallbladder (ESGE Grade: severe)
	Subsequent surgical intervention	Elective cholecystectomy	Surgery not planned due to age and comorbidities	Urgent open cholecystectomy with foreign body removal
	Clinical outcome	Uneventful recovery; inflammation resolved with antibiotics; discharged	Uneventful recovery; antibiotic therapy continued; discharged	Uneventful postoperative recovery; discharged

AF, atrial fibrillation; CBD, common bile duct; CKD, chronic kidney disease; CT, computed tomography; DM, diabetes mellitus; ERCP, endoscopic retrograde cholangiopancreatography; ESGE, European Society of Gastrointestinal Endoscopy; MDT, multidisciplinary team; MRCP, magnetic resonance cholangiopancreatography; N/A, not applicable; SIT, situs inversus totalis; TG18, Tokyo Guidelines 2018.
